# Home Health Access among Persons with Dementia: Traditional Medicare vs. Medicare Advantage

**DOI:** 10.1093/geroni/igaf122.3601

**Published:** 2025-12-31

**Authors:** Chenjuan Ma

**Affiliations:** New York University, New York, New York, United States

## Abstract

Home health care (HHC) is the most used home and community-based service for community-dwelling Americans, particularly persons living with dementia. There are two ways of accessing HHC: entry directly from the community or following institutional care (primarily after hospitalization for acute care). This study compared HHC access (community-entry vs. hospital entry) between persons with and without dementia and examined the role of Medicare Advantage (MA) HHC access. It used 2019 national home health assessment data (i.e., OASIS) linked to the Medicare Beneficiary Summary File. Multivariate logistic regression was used in the analysis, adjusted for patient sociodemographic and clinical conditions, and considering clustering within each agency. Of the 3,174,874 home health episodes in 2019, 42% were community-entry HHC, 22% were from persons with dementia, and 35% were from MA beneficiaries. Persons with dementia were more likely to be traditional Medicare (TM) beneficiaries (31% TM with dementia vs. 6% MA with dementia). Persons with dementia were significantly more likely to enter HHC directly from the community (60% vs. 37%, adjusted OR = 1.52, 95% CI = 1.50-1.53). Among persons with dementia, having MA was less likely to enter HHC from the community (adjusted OR = 0.93, 95% CI = 0.90-0.95), compared to having TM; while among persons without dementia, having MA was more likely to enter HHC from the community (adjusted OR = 1.08, 95% CI = 1.06-1.11). This study indicates the importance of community-entry HHC for high-need populations such as persons with dementia. It highlights the need for continuing monitoring of community-entry HHC accessibility under a rapidly changing healthcare system.

